# Changes in physical activity during a one-year weight loss trial with liraglutide vs placebo in participants with knee osteoarthritis: Secondary analyses of a randomised controlled trial

**DOI:** 10.1016/j.ocarto.2022.100255

**Published:** 2022-03-18

**Authors:** Cecilie Bartholdy, Anders Overgaard, Henrik Gudbergsen, Henning Bliddal, Lars Erik Kristensen, Marius Henriksen

**Affiliations:** The Parker Institute, Bispebjerg-Frederiksberg Hospital, University of Copenhagen, Frederiksberg, Denmark

**Keywords:** Physical activity, Knee osteoarthritis, Weight loss, Liraglutide

## Abstract

**Objective:**

To assess if a change in physical activity occurred after a one-year weight loss period on either liraglutide or placebo in patients with knee osteoarthritis (OA) and overweight.

**Method:**

This is secondary analysis of a one-year weight loss trial, with participants randomised (1:1) to either liraglutide 3 ​mg/day or placebo. The main outcome was change in physical activity (min/day) after one year assessed by accelerometer. Physical function was assessed using the Knee Injury and Osteoarthritis Outcome Score (KOOS), function subscale with 100 indicating no disability and 0 indicating extreme disability. Analyses were done on the modified intention to treat population defined as complete baseline accelerometer data.

**Results:**

A total of 135 participants were analysed (66 liraglutide; 69 placebo). Daily physical activity time did not change in either group (liraglutide: 15.8 ​min/day; placebo: 14.2 ​min/day; mean difference 1.6 ​min/day (95%CI -16 to 19; P ​= ​0.90)). The liraglutide group lost −4.1 ​kg more than placebo (95% CI -6.0 to −2.1; P ​< ​0.0001) and improved in KOOS function 3.8 points more than placebo (95% CI 0.9 to 6.7; P ​= ​0.01).

**Conclusion:**

Despite better outcomes on body weight and self-reported physical functioning liraglutide did not induce changes in physical activity over one year in individuals with knee OA.

## 1 Introduction

Lack of physical activity (moderate-to-vigorous) or high amount of sedentary time (sitting/reclined) have detrimental effects on overall health [1]. People with disability, such as patients with knee osteoarthritis (OA), are at increased risk of spending too little time on physical activity [2], and the combination of disability and sedentary behaviour increase the risk of cardiovascular disease and mortality [3]. Further, many patients with knee OA are overweight or obese, which associates with both disability and with physical inactivity [4] Weight loss is recommended for overweight individuals with knee OA [5], and weight loss significantly improves knee OA disability [6]. Further, overweight has been identified as a modifiable factor that associates with physical inactivity in knee OA, and weight loss has been suggested to be important to address if physical activity is to be increased among individuals with knee OA [7].

Weight loss is traditionally achieved through dietary and behavioural changes. However, recently, pharmacological agents, such as liraglutide (a GLP-1 receptor antagonist), have shown reliable and relevant weight loss in knee OA [8]. It is, however, unknown if a pharmaceutically induced weight loss (by liraglutide) has spontaneous secondary beneficial effects on physical activity, or if additional measures are needed to achieve this. Accordingly, the purpose of this study was to compare spontaneous changes in physical activity after a one-year weight loss period with either liraglutide or placebo in overweight persons with knee OA.

## 2 Methods

This study was part of a randomised trial “*Effect of liraglutide on body weight and pain in overweight or obese patients with knee osteoarthritis*” (clinicaltrials.gov; NCT02905864) [8]. All participants partook in an 8-week run-in dietary intervention period where they followed an intensive dietary intervention (IDI) of 800–1000 ​kcal a day to produce a weight loss of minimum 5%. Participants, who achieved at least 5% weight loss, were included in the one-year randomised trial, in which they were randomly allocated (1:1) to either liraglutide 3 ​mg/day or placebo, stratified based on sex (female vs. male), age (<60 years vs. ​≥ ​60 years), and obesity class (BMI <40 ​kg/m2 vs. ​≥ ​40 ​kg/m2). All staff involved in tests of participants or reporting of data were blinded.

### 2.1 Inclusion/exclusion criteria

The main inclusion criteria were: clinical diagnosis of knee OA (American College of Rheumatology criteria) confirmed by radiology (Kellgren-Lawrence score 1, 2 or 3), age 18 to 74, BMI ≥27 ​kg/m2 (for inclusion in the IDI period), motivated for weight loss, minimum 5% weight loss after completing the 8-week IDI period. Individuals with ongoing or recent participation in organized weight loss programs, current or recent use of weight loss medications, and end-stage knee OA on radiography (Kellgren-Lawrence score ​= ​4) were ineligible for participation. Participants were recruited from the Osteoarthritis outpatients clinic, Frederiksberg Hospital, Denmark.

### 2.2 Study design

In the 1-year randomised trial, liraglutide or placebo was administered subcutaneously starting with 0.6 ​mg/day increasing biweekly by 0.6 ​mg/day until 3 ​mg/day was reached. During the first 8 weeks of the 1-year period both groups had biweekly sessions with a dietitian to re-introduce regular meals in combination with meal replacement products. For details, see Ref. [8].

### 2.3 Outcomes

The main outcome for this study was change in physical activity (min/day) from randomization to one-year. An accelerometer with an inbuilt thermometer (dimensions: 50x21 ​× ​5 mm, weight: 8g, SENS-MOTION® activity measurement system, version 1.7.1) placed on the thigh was used to measure physical activity continuously (24h/day) one week before randomization and at the one-year assessment. The assessment of physical activity comprised of all movements e.g. excluding time spent standing or sitting/laying down (physical inactivity). The accelerometer data was considered valid if at least 24-h wear time was recorded; this was determined by inspection of the temperature recordings. Physical activity was assessed the average time related to the total wear time and expressed as minutes/day. The validity and reliability of the accelerometer and the inbuilt algorithm for detecting physical activity and sedentary behaviour in persons with knee OA has been established as satisfactory [9].

Disability was assessed by the function subscale of the Knee Injury and Osteoarthritis Outcome Score (KOOS). The KOOS function subscale consists of 17 items related to physical function in daily life. Each item is scored on a 5-point Likert scale scoring from 0 to 4, and the subscale is normalized to a 0–100 scale with 100 indicating no disability in daily life and 0 indicating extreme disability.

### 2.4 Statistical analysis

The analysed population consisted of participants with valid accelerometer data at baseline (modified intention-to-treat population). To assess the difference between groups an ANCOVA model was applied with group as fixed factor, adjusted for stratification factors; sex, age category (age ≤60), obesity class (BMI ≤40), and baseline value. Missing data at one-year was dealt with using baseline observation carried forward.

## 3 Results

**Fig. 1:**
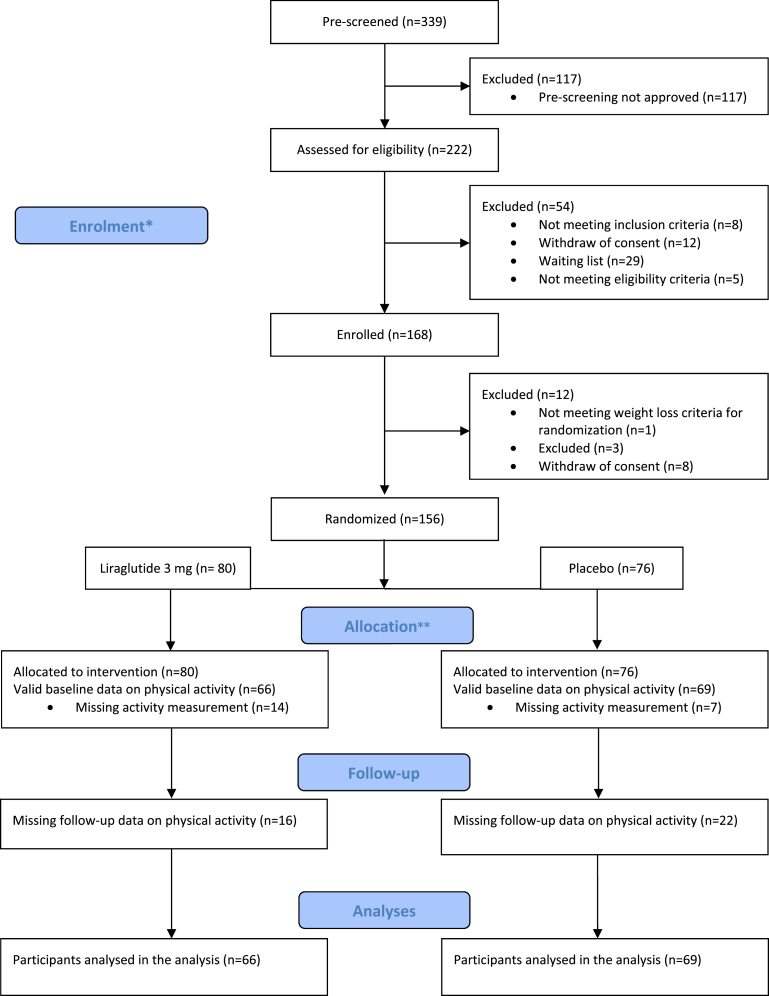
Flow chart of participants in the trial. ∗ Patients were enrolled in an intensive run-in dietary intervention period (week −8 to 0) that included a low-calorie diet from Cambridge Weight Plan and dietetic counselling. ∗∗ Upon successfully achieving a weight loss of minimum 5% at week 0, patients continued with a tapering dietary intervention for 8 weeks (week 0–8) and were randomised in a 1:1 manner to receive either liraglutide (3 ​mg/day) or identically appearing placebo throughout the 52-week main study period (week 0–52).

A total of 156 participants were randomised to either liraglutide (n ​= ​80) or placebo (n ​= ​76), of those 135 participants had valid baseline accelerometer data (66 liraglutide and 69 placebo) (see Fig. 1). At baseline the median accelerometer wear time was 7 days (mean: 5.5 days; range 2–7 days); at the 1-year assessment the median was 7 days (mean: 6.4 days; range 2–7 days). For the liraglutide group the average age was 58.8 years, height 171.5 ​cm and BMI 33 ​kg/m^2^. In the placebo group the average age was 58.6 years, height 170.4 ​cm and BMI 31.5 ​kg/m^2^.

Table 1 Presents the baseline values, changes within groups, and difference between groups in body weight, KOOS Function, and physical activity measures. There were statistically significant differences between groups in terms of weight loss and change in KOOS function of 3.8 points favouring liraglutide. The group difference in changes in the physical activity measures were not significantly different between groups (Table 1).

**Table 1:** Baseline and follow-up data for each group in the modified intention to treat population (n ​= ​135), defined as participants with valid baseline accelerometer data. Baseline data are presented as mean with standard deviation (SD); changes within each group is presented as mean and standard error (SE), and difference between groups are presented with mean, 95% confidence interval (95% CI) and p value.

	Baseline		Change after one year				
	Mean (SD)		Mean change (SE)		Difference between groups		
	Liraglutide (n ​= ​66)	Placebo (n ​= ​69)	Liraglutide (n ​= ​66)	Placebo (n ​= ​69)	Mean	95% CI	P value
Age	58.8 (11.3)	58.6 (9.6)	–	–	–	–	–
Female sex, n (%)	43 (65.1%)	44 (63.8%)	–	–	–	–	–
BMI	33.0 (5.9)	31.5 (4.1)	–	–	–	–	–
Kellgren Lawrence score							
1, n (%)	9 (13.6%)	12 (17.4%)	–	–	–	–	–
2, n (%)	27 (40.9%)	31 (44.9%)	–	–	–	–	–
3, n (%)	30 (45.5%)	26 (37.7%)	–	–	–	–	–
Comorbid diabetes, n (%)	7 (10.6%)	4 (5.8%)	–	–	–	–	–
Weight, kg	97.1 (19.0)	91.6 (14.4)	−6.4 (1.1)	−2.3 (1.2)	−4.1	−6.0 to −2.1	<0.0001
KOOS Function, 0-100	81.0 (15.1)	85.1 (10.7)	3.7 (1.5)	−0.1 (1.6)	3.8	0.9 to 6.7	0.01
Physical activity, min/day	234.4 (71.6)	235.4 (85.6)	15.8 (8.7)	14.2 (9.4)	1.6	−16.0 to 19.1	0.9
Resting, min/day	1067.7 (112.0)	1052.0 (124.2)	0.6 (10.7)	5.8 (11.5)	−5.2	−26.8 to 16.4	0.6
Standing, min/day	123.6 (58.1)	138.2 (93.2)	−3.5 (6.5)	−9.4 (7.0)	5.9	−7.2 to 19.0	0.4

## 4 Discussion

The purpose of this study was to compare changes in physical activity after a one-year weight loss period with between treatment with liraglutide or placebo in overweight people with knee OA. Overall, the majority of time was spent in resting (standing, sitting or lying) and the time spent on physical activity did not change significantly during a one-year weight loss period in either group, despite that the liraglutide group had a significantly greater weight loss and slightly improved physical function compared to the placebo group.

The present results corroborate the results from the 8-week IDI period [10] in which time spent on physical activity was unchanged despite a large weight loss (12.7 ​kg) and changes in self-reported function (mean change 14.5 points). The same observation has been seen following an 8 week exercise and education program [11]. The lack of change in time spent on physical activity following the 1-year weight loss trial could be because of the small and clinically irrelevant change in self-reported function at 1 year and the relatively high baseline scores indicating only marginal baseline disability. Our results are limited by the fact that we did not measure the intensities of the physical activity, which may have changed. Nevertheless, both groups spent less time being physically active than what is recommended for an overall healthy lifestyle.

An explanation for the lack of change in overall time spent on physical activity might be that the intensity of the activities performed changed but not the overall time. This was not assessed in this study as the accelerometer algorithm is not validated for such analyses [9]. If such a change occurred, it would give the participants an added health benefit. Nevertheless, as a group they spent less time being physically active than recommended for an overall healthy lifestyle [12].

Changing this population's physical activity behaviour in combination with or after a weight loss is relevant for these patients, as they still contribute to the overall burden on the health care system [13]. However, these and other studies suggest that this is unlikely to occur spontaneously following primary treatment of knee OA symptoms [10,11]. The findings are supported by other studies finding small relation between knee OA symptoms [12] and physical activity. Even after total knee replacement no spontaneous change in physical activity occur [14]. Hence, changing physical activity behaviour in patients with knee OA seems to go beyond changing the primary symptoms (pain and disability) and overweight. Rather specific psychological and behavioural motivational interventions seem necessary [15].

In conclusion, time spent in physical activity is unaffected by a long-term weight loss intervention by liraglutide compared to placebo. Targeted treatment specifically aiming at changes in physical activity seems necessary.

## Funding

The main trial was funded by NOVO Nordisk A/S and Cambridge Weight Plan. The Parker Institute is supported by an unspecific grant from the 10.13039/100001275Oak Foundation (OCAY-13-309). The funding partners had no part in the planning, execution and interpretation of this study.

## Credit author statement

CB, MH, conceptualized the study. CB, MH, AO, LEK, HG, HB, took part in writing, reviewing, and approving the manuscript. CB, MH performed the data capture and analysis. CB, AO, HB acquisitioned clinical data. All authors read and approved the final manuscript.

## Ethical approval information

The protocol was approved by the local health research ethics committee (H-16019969).

## Data sharing statement

Data are available upon reasonable request.

## Declaration of competing interest

None.
